# Molecular epidemiology of salmonid alphavirus (SAV) subtype 3 in Norway

**DOI:** 10.1186/1743-422X-7-188

**Published:** 2010-08-11

**Authors:** Mona D Jansen, Britt Gjerset, Ingebjørg Modahl, Jon Bohlin

**Affiliations:** 1 Center for Epidemiology and Biostatistics, Norwegian School of Veterinary Science, Oslo, Norway; 2 Section for Virology, National Veterinary Institute, Oslo, Norway

## Abstract

**Background:**

Pancreas disease (PD) is a viral fish disease which in recent years has significantly affected Norwegian salmonid aquaculture. In Norway, the aetiological agent salmonid alphavirus (SAV) has been found to be represented by the subtype 3 only. SAV subtype 3 has in previous analyses been found to show a lower genetic divergence than the subtypes found to cause PD in Ireland and Scotland. The aim of this study was to evaluate the nucleotide (nt) and amino acid divergence and the phylogenetic relationship of 33 recent SAV subtype 3 sequences. The samples from which the sequences were obtained originated from both PD endemic and non-endemic regions in an attempt to investigate agent origin/spread. Multiple samples throughout the seawater production phase from several salmonid populations were included to investigate genetic variation during an outbreak. The analyses were mainly based on partial sequences from the E2 gene. For some samples, additional partial 6 K and nsP3 gene sequences were available.

**Results:**

The nucleotide divergence for all gene fragments ranged from total identity (0.0% divergence) to 0.45% (1103 nt fragment of E2), 1.11% (451 nt fragment of E2), 0.94% (6 K) and 0.28% (nsP3). This low nucleotide divergence corresponded well to previous reports on SAV 3 sequences; however the observed divergence for the short E2 fragment was higher than that previously reported. When compared to SAVH20/03 (AY604235), amino acid substitutions were detected in all assessed gene fragments however the *in vivo *significance of these on for example disease outbreak mortality could not be concluded on. The phylogenetic tree based on the 451 nt E2 fragment showed that the sequences divided into two clusters with low genetic divergence, representing only a single SAV subtype.

**Conclusions:**

The analysed sequences represented two clusters of a single SAV subtype; however some of the observed sequence divergence was higher than that previously reported by other researchers. Larger scale, full length sequence analyses should be instigated to allow further phylogenetic and molecular epidemiology investigations of SAV subtype 3.

## Background

The fish disease known as pancreas disease (PD) impacts significantly on Norwegian salmonid aquaculture, affecting both Atlantic salmon (*Salmo salar *L.) and rainbow trout (*Oncorhynchus mykiss*) seawater production[1-3]. In addition, Scottish and Irish Atlantic salmon production has been severely affected since the emergence of PD in Scotland in 1976 [4,5]. High proportions of the salmonid aquaculture sites have been continually affected by PD in both Ireland and Scotland; with Irish figures estimating 95% of examined Irish farms affected by PD between 1985 and 1989 [6], 62% affected in 2003 and 86% in 2004[7]. PD emerged in Norwegian aquaculture in the 1980s [8], followed by a gradual increase in the number of cases diagnosed within two western counties (Hordaland and Sogn & Fjordane) initially constituting the endemic region. A gradual expansion of this endemic region southwards (Rogaland, 2004) and northwards (Møre & Romsdal, 2006) resulted in almost the entire south-western coast constituting an endemic region by the end of 2006. The first cases outside this region were detected in 2003 in the two northernmost counties (Finnmark and Troms), with Troms also affected in 2009. An area within the northernmost county (Finnmark) may be considered to constitute a separate endemic area, having had one or two cases diagnosed each year between 2005 and 2008. A third, northern county has been affected (Nordland, 2004 and 2008), as well as one county in mid-Norway (Sør Trøndelag, 2009). The number of Norwegian seawater sites with diagnosed or suspected PD peaked at 109 in 2008, while declined to 75 in 2009 following industry and government efforts to reduce the impact of the disease. Although having a serious impact on Norwegian salmonid aquaculture, the proportion of affected sites in Norway remains lower than that seen in the Scottish and Irish industries. PD-affected fish generally show anorexia and lethargy, and develop SAV-associated lesions particularly in exocrine pancreas and heart- and skeletal muscle [3]. PD-associated mortality levels vary greatly, with a range between 0.7 and 26.9% seen in recently studied Norwegian sites [9].

The aetiological agent was first isolated in Ireland [10] and was later identified as an alphavirus in the family *Togaviridae *[11,12]. The species name salmonid alphavirus (SAV) was suggested [12] and has been adopted by researchers despite not being accepted by the International Committee on Taxonomy of Viruses. The SAV nomenclature will be used throughout this paper. Six SAV subtypes have been classified so far. In Ireland SAV subtypes 1, 4, and 6 have been isolated from fish affected by PD, while Scottish outbreaks have been caused by SAV subtypes 1, 2, 4, and 5[13,14]. From Norwegian PD outbreaks, only SAV subtype 3 has been detected [2,13-15], with a very low level of genetic variance between isolates [13,15]. Although now isolated from Atlantic salmon in the seawater phase [13]; the majority of outbreaks due to SAV subtype 2 occurs in freshwater farms stocking rainbow trout where the resultant disease has become known as sleeping disease (SD) [16]. As with other alphaviruses, SAV has a positive sense, single stranded RNA genome [17] of approximately 12 kb [12]. The non-structural proteins (nsP1 to nsP4) are encoded by the 5' end and the structural proteins (capsid, envelope glycoproteins (E1 to E3) and 6 K) by the 3' end [17]. The alphavirus structural protein E2 has been found to be the site of most neutralising epitopes [18]. Salmonid alphaviruses have been found to be genetically distinct from the other alphaviruses, many of which use arthropod vectors in their transmission [18]. No vectors have been found to be included in SAV transmission, and horizontal transmission pathways appear to be most important for the spread of SAV and PD between seawater populations [2,7-9,13-15,19-25].

The aim of this study was to evaluate the nucleotide (nt) and amino acid divergence as well as the phylogenetic relationship of 33 recently obtained SAV subtype 3 sequences originating from both PD endemic and non-endemic regions of Norway. Based on the results, the possibility of gaining information on agent origin/spread were to be investigated. Multiple samples throughout the seawater production phase from several salmonid populations were included to investigate the presence of genetic changes during an outbreak. Analyses were to be based mainly on the partial E2 gene, with additional partial 6 K and nsP3 gene sequences available from some samples.

## Methods

### Sample selection

Samples originated from SAV-positive Atlantic salmon in the seawater production phase. A total of 33 SAV-positive samples from 12 seawater sites were selected for partial sequence analysis (Table 1). Multiple samples, originating from one to three sampling points, were included from nine sites. The sampling point(s) at each site varied in time, ranging from two months post seawater transfer to slaughter. As a result, almost the entire seawater production cycle was represented and gave a wide range in fish age and weight at time of sampling. Samples from six sites located within the endemic region were selected from participants in a cohort study [9]. Out of these, four sites (sites 1, 3, 4 and 6, Table 1) were included as they were found SAV-positive earlier in the seawater phase than the majority of the studied sites. Further four diagnostic samples from site 3, from an outbreak investigation on the fish generation put to sea after the slaughter of the cohort study generation, were included. Samples from two additional cohort study sites were included as they represented the minimum (site 5, Table 1) and maximum (site 2, Table 1) recorded PD-associated mortality. Finally, diagnostic samples submitted from six sites (sites 7 to 12, Table 1) in the non-endemic region or the endemic area of Finnmark in December 2003 or between November 2007 and October 2009 were included. All selected sites had PD diagnosed by SAV detection by real-time RT-PCR (Rt RT-PCR) being combined with histopathological changes in accordance with PD (as described by [9]).

**Table 1:** Isolate identification, accession numbers and additional data for 33 SAV 3 study isolates

Isolate identification	Site	Region (Endemic/Non-endemic)	County	Sample month & year	Sample origin	PD outbreak peak mortality (%)	E2 + 6 K (*) or E2 sequence length (nt)	Accession number E2/6 K or E2	Accession number nsP3
SAVH06-1(1)	1	Endemic	Hordaland	Sep 2006	Cohort	2.6	451	HM208094	n/a
SAVH06-1(2)	1	Endemic	Hordaland	Sep 2006	Cohort	2.6	1209*	HM208095	n/a
SAVH07-1(3)	1	Endemic	Hordaland	Jan 2007	Cohort	2.6	1209*	HM208096	n/a
SAVH07-2(1)	2	Endemic	Hordaland	Nov 2007	Cohort	26.9	451	HM208097	n/a
SAVH07-2(2)	2	Endemic	Hordaland	Nov 2007	Cohort	26.9	451	HM208098	n/a
SAVH07-3(1)	3	Endemic	Hordaland	Nov 2007	Cohort	12.2	451	HM208099	n/a
SAVH07-3(2)	3	Endemic	Hordaland	Nov 2007	Cohort	12.2	451	HM208100	n/a
SAVH07-3(3)	3	Endemic	Hordaland	Nov 2007	Cohort	12.2	451	HM208101	n/a
SAVH07-3(4)	3	Endemic	Hordaland	Nov 2007	Cohort	12.2	1209*	HM208102	HM208125
SAVH07-3(5)	3	Endemic	Hordaland	Nov 2007	Cohort	12.2	451	HM208103	n/a
SAVH09-3(6)	3	Endemic	Hordaland	Jul 2009	Outbreak	n/a	451	HM208104	n/a
SAVH09-3(7)	3	Endemic	Hordaland	Jul 2009	Outbreak	n/a	451	HM208105	n/a
SAVH09-3(8)	3	Endemic	Hordaland	Jul 2009	Outbreak	n/a	451	HM208106	n/a
SAVH09-3(9)	3	Endemic	Hordaland	Jul 2009	Outbreak	n/a	451	HM208107	n/a
SAVSF06-4(1)	4	Endemic	Sogn og Fjordane	Aug 2006	Cohort	5.2	1209*	HM208114	HM208129
SAVSF07-4(2)	4	Endemic	Sogn og Fjordane	Apr 2007	Cohort	5.2	1170*	HM208115	n/a
SAVSF07-4(3)	4	Endemic	Sogn og Fjordane	Oct 2007	Cohort	5.2	1209*	HM208116	n/a
SAVSF07-4(4)	4	Endemic	Sogn og Fjordane	Oct 2007	Cohort	5.2	1209*	HM208117	n/a
SAVMR07-5(1)	5	Endemic	Møre og Romsdal	Jan 2007	Cohort	0.7	1209*	HM208108	HM208126
SAVMR07-5(2)	5	Endemic	Møre og Romsdal	Jan 2007	Cohort	0.7	451	HM208109	n/a
SAVMR07-6(1)	6	Endemic	Møre og Romsdal	Feb 2007	Cohort	11.5	1209*	HM208110	HM208127
SAVMR07-6(2)	6	Endemic	Møre og Romsdal	Nov 2007	Cohort	11.5	1203*	HM208111	n/a
SAVST09-7(1)	7	Non-end	Sør Trøndelag	Apr 2009	Outbreak	n/a	1209*	HM208118	n/a
SAVST09-7(2)	7	Non-end	Sør Trøndelag	Apr 2009	Outbreak	n/a	1209*	HM208119	n/a
SAVST09-7(3)	7	Non-end	Sør Trøndelag	Apr 2009	Outbreak	n/a	1209*	HM208120	n/a
SAVST09-7(4)	7	Non-end	Sør Trøndelag	Apr 2009	Outbreak	n/a	1209*	HM208121	n/a
SAVN03-8(1)	8	Non-end	Nordland	Dec 2003	Outbreak	n/a	1103	HM208112	HM208128
SAVN08-9(1)	9	Non-end	Nordland	Jul 2008	Outbreak	n/a	451	HM208113	n/a
SAVT09-10(1)	10	Non-end	Troms	Oct 2009	Outbreak	n/a	451	HM208122	n/a
SAVT09-10(2)	10	Non-end	Troms	Oct 2009	Outbreak	n/a	451	HM208123	n/a
SAVF07-11(1)	11	Endemic^1^	Finnmark	Nov 2007	Outbreak	n/a	451	HM208091	n/a
SAVF07-11(2)	11	Endemic^1^	Finnmark	Nov 2007	Outbreak	n/a	451	HM208092	n/a
SAVF08-12(1)	12	Endemic^1^	Finnmark	Jan 2008	Outbreak	n/a	1209*	HM208093	HM208124

Isolate identification has been generated as follows: SAV, initial(s) of the originating county, year of sampling - site number (isolate number for site).

* sequence includes partial 6 K sequence

n/a not available

^1 ^constitutes a separate endemic area with repeated outbreaks in the north of the non-endemic area

### RNA extraction and Rt RT-PCR

RNA was extracted from a mixture of heart and mid-kidney tissue according to the protocol previously described [9]. A 1762 base pair (bp) region within the nsP3 gene and a 1871 bp region within the Capsid-E3-E2-6 K genes, corresponding to positions 4206-5968 and 8411-10282 of the Norwegian SAVSF21/03 (AY604238) respectively, were amplified using partial overlapping sequences. For the Capsid-E3-E2-6 K genes three primer pairs were used, with two primer pairs used for the nsP3 gene (Table 2). The primer sequence of F1600, R2357, F2234 and SAV20R originated from work by other researchers [15]. Briefly, extracted RNA was reverse transcribed using random primers and SuperScript III RT (Invitrogen) or OneStep (Qiagen); 2.5 μl cDNA with 0.15 μM of each primer were added in a final PCR reaction volume of 25 μl (HotStar Taq PCR; Qiagen) under the following conditions: denaturation for 15 minutes at 95°C, followed by 40 amplification cycles of 94°C 30 sec, 59°C 30 sec and 72°C 90 sec, and finally 72°C for 10 min. The RT-PCR products were examined by agar gel electrophoresis and purified using the ExoSAP-IT protocol (Usb) prior to sequencing with BigDye^® ^Terminator v3.1 Cycle Sequencing Kits (Applied Biosystems).

**Table 2:** Capsid-E3-E2-6K and nsP3 primer pair details

Gene fragment	Forward primer	Forward primer sequence	Reverse Primer	Reverse primer sequence
C-E3-E2-6K	F1600	CGGCACTATCAGAGTGGAGGA	R2375	AGGATGTAGTGGCCGGTGG
C-E3-E2-6K	F2234	CGGGTGAAACATCTCTGCG	SAV20R	GGCATTGCTGTGGAAACC
C-E3-E2-6K	E2666F	GCGACCGTTACCTTTACCAGCG	E2YR	CAGCACAGTCTGCAGTGTCTAAG
nsP3	nsP3YF	GAAAGTGGCGGAGATCCTCA	nsP3940R	TGAGCGGCAGTTTGAATGC
nsP3	nsP3930F	ACTGCCGCTCACTAACATCCA	nsP3YR	GGGTATTATGCTGGCTAAGGTGAG

### Sequence analysis

Consensus sequences were generated using Sequencher (Gene Codes Corporation) or ChromasPro (Technelysium Pty Ltd). All sequences were edited so that the longest shared and least conserved genetic region was included in the analysis. All sequence analyses and editing was carried out with the aid of the MEGA4 software [26]. After editing, 13 sequences contained a 1209 bp fragment corresponding to position 9049-10257 of the Norwegian SAV SF21/03 isolate (AY604238), covering a major part of the E2 gene together with a portion of the 6 K gene. An additional three sequences covered slightly shorter fragments (SAVMR07-6(2): 1203 bp, position 9049-10251; SAVSF07-4(2): 1170 bp, position 9049-10218; SAVN03/8(1): 1103 bp, position 9049-10151(E2 only)) (Table 1). Further 17 sequences covered a 451 bp E2 fragment corresponding to position 9224-9674 (SAV SF21/03, AY604238), while six sequences covered a 716 bp fragment of the nsP3 region corresponding to position 5183-5898 (SAV SF21/03, AY604238) (Table 1). The sequences were aligned using both Muscle [27] and Clustal [28]. Pair wise nucleotide percentage similarity and divergence was calculated using the program Laglin (available at http://www.ch.embnet.org/index.html). Phylogenetic trees were generated from the multiple alignments using maximum parsimony (MP), unweighted pair group method using arithmetic average (UPGMA) and neighbor joining (NJ) methods, and generated using both the MEGA4 and Seaview (version 4) software packages [29]. Sequence data from eight SAV subtype 3 were obtained from GenBank and included in the phylogenetic analyses (SAVH20/03 (AY602435), SAVH10/02 (AY604236), PD97-N03 (AY604237), SAVSF21/03 (AY604238), SAVF29/03 (DQ122127), SAVT28/03 (DQ122128), SAVN32/04 (DQ122129), SAVSF22/03 (DQ122131)). Additionally, the Irish SAV 1 reference strain F93-125 (AJ316244) and the French SAV 2 reference strain S49p (AJ316246) were included. The phylogenetic tree shown in this paper was based on the NJ method and bootstrapped 1000 times. The 33 study sequences are available from GenBank, with accession numbers as shown in Table 1.

## Results

### E2 and 6 K fragment

Amongst the 16 sequences covering the 1103 nt E2 fragment, the nucleotide divergence ranged from 0.0% to 0.45%. When compared to SAVH20/03 (AY604235), amino acid substitutions were detected in two sequences (Table 3). In the 106 nt 6 K fragment, a nucleotide divergence between 0.0% and 0.94% was found with five sequences showing an amino acid substitution (Table 3). The 17 sequences covering the shorter, 451 nt E2 fragment had a nucleotide divergence between 0.0% and 1.11%. Amino acid substitutions were observed in 12 sequences (Table 3).

**Table 3:** Amino acid substitutions in SAV subtype 3 study sequences relative to the reference SAVH20/03 (AY604235)

Gene/Position	E2/153	E2/185	E2/190	E2/204	E2/206	E2/229	6K/8	nsP3/415	nsP3/425	nsP3/536	E2 length (nt)
Isolate											
SAVH20/03	K	L	I	R	S	S	I	I	T	S	1103
SAVF07-11(1)	R	*	*	K	P	G	-	-	-	-	451
SAVT09-10(1)	*	M	*	K	P	*	-	-	-	-	451
SAVH07-2(1)	*	*	T	*	*	*	-	-	-	-	451
SAVH07-2(2)	*	*	T	*	*	*	-	-	-	-	451
SAVF08-12(1)	*	*	*	K	P	G	*	*	*	*	1103
SAVF07-11(2)	*	*	*	K	P	G	-	-	-	-	451
SAVH07-3(1)	*	*	*	K	P	G	-	-	-	-	451
SAVH07-3(2)	*	*	*	K	P	G	-	-	-	-	451
SAVT09-10(2)	*	*	*	K	P	*	-	-	-	-	451
SAVH09-3(8)	*	*	*	K	P	*	-	-	-	-	451
SAVH09-3(9)	*	*	*	K	P	*	-	-	-	-	451
SAVH07-3(3)	*	*	*	K	P	*	-	-	-	-	451
SAVH07-3(4)	*	*	*	K	P	*	*	V	A	*	1103
SAVH06-1(1)	*	*	*	*	*	N	-	-	-	-	451
SAVST09-7(1)	*	*	*	*	*	*	T	*	*	*	1103
SAVST09-7(2)	*	*	*	*	*	*	T	*	*	*	1103
SAVST09-7(3)	*	*	*	*	*	*	T	*	*	*	1103
SAVST09-7(4)	*	*	*	*	*	*	T	*	*	*	1103
SAVMR07-5(1)	*	*	*	*	*	*	T	*	*	*	1103
SAVSF06-4(1)	*	*	*	*	*	*	*	*	*	T	1103

Only study sequences showing amino acid substitutions relative to SAVH20/03 has been included, and has been tabulated in the order in which the amino acid substitutions occur.

* amino acid identical to reference SAVH20/03

- sequence not available for the isolate

### nsP3 fragment

In the six sequences covering the 716 nt partial nsP3 fragment the nucleotide divergence ranged from 0.0% and 0.28%. Amino acid substitution(s) were detected in two sequences (Table 3), one of which also showed an amino acid substitution in the E2 fragment.

### Phylogenetic analyses

**Figure 1:**
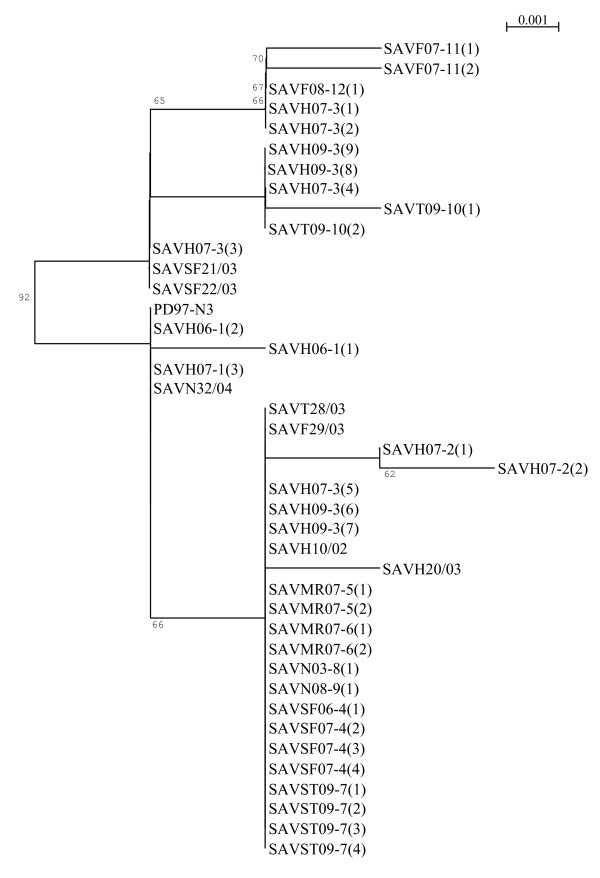
**Phylogenetic tree based on 33 SAV subtype 3 study sequences and eight GenBank obtained sequences**. The phylogenetic tree (NJ method, bootstrapped 1000 times) was based on a 451 nt E2 sequence. Bootstrap-values above 60 have been displayed.

Both sequence-alignment programs and all three tree-generation methods produced identical results. Three phylogenetic trees were generated based on the nucleotide sequences of the obtained isolates; E2-6K sequences, short E2 sequences and nsP3 sequences. All three trees showed similar topology. The tree covering the largest number of sequences, 33 sequences covering the 451 nt (short) E2 fragment, has been included in this paper (Figure 1). The Irish SAV 1 reference strain F93-125 (AJ316244) and French SAV 2 reference strain S49p (AJ316246) formed completely separate clusters from the Norwegian sequences (bootstrap 100) in all generated trees and has not been displayed.

## Discussion

The observed nucleotide divergence amongst our study sequences was generally low, with the short E2 sequences showing the highest divergence (up to 1.1%); followed closely by the 6 K sequences (up to 0.94%). The longer sequences showed a lower divergence, with the long E2 sequences showing a slightly higher divergence (up to 0.45%) than the nsP3 sequences (up to 0.28%). The low divergence amongst our Norwegian sequences corresponded well with that reported from previous analyses of SAV subtype 3 sequences; however the divergence amongst our short E2 sequences was higher than that previously reported [13,15]. The divergence seen amongst the short E2 and 6 K sequences may be artificially inflated to some degree by covering only a relatively small portion of the respective genes, which may represent the most variable region within these. On the other hand, it may be that this within-subtype variance is a true representation of the current SAV subtype 3 affecting Norwegian aquaculture. The sequences included in this study were, with one exception, covering August 2006 to October 2009, and originated from affected populations both inside and outside the endemic region. Our analysis, covering a total of 33 Norwegian SAV subtype 3 sequences, is the largest reported analysis of Norwegian sequences and covered more recent sequences than those previously published. SAV subtypes originating in Ireland and Scotland have been reported to show higher nucleotide divergence than SAV subtype 3 (SAV subtypes 1, 2, 4, and 5: E2 fragment divergence 1.2%, 4.8%, 3.4% and 1.7%; nsP3 fragment divergence 0.8%, 6.6%, 3.7% and 4,2%) [13]. RNA viruses are generally rapidly evolving viruses; however alphaviruses, including SAV, appears to be comparatively highly conserved with slower rates of evolution [30-32]. It is possible that the observed difference in within-subtype nucleotide divergence of SAV subtype 3 and the other SAV subtypes can be related to the differences in the proportion of susceptible populations (sites) affected in Norway compared to Ireland and Scotland. Based on the published reports, PD also appears to have been present in Scottish aquaculture for a longer time period. In Norway the proportion of affected populations remain well below that seen in Ireland and Scotland, were the majority of susceptible populations are affected. This difference, together with the historical differences in emergence of PD, may have resulted in differing evolutionary pressure on the respective SAV subtypes. It is possible that a continued high impact on Norwegian aquaculture, with or without a further expansion in geographical distribution, may result in a gradual increase in the sequence divergence towards that of other SAV subtypes. Our results support the theory that there has been only a single introduction of SAV subtype 3 into Norwegian aquaculture, from which it has dispersed to reach its current distribution.

The observed amino acid substitutions were partially the same as those previously reported in SAV subtype 3. Similar substitutions to those reported at E2 position 204 (R to K) and 206 (S to P) [15] was seen in 11 of our sequences originating from four sites. *In vitro *studies have reported this serine to proline substitution at position 206 to be associated with the appearance of a cytopathic effect [15]. The *in vivo *significance of this substitution remains unclear. It was only possible to obtain reliable data on the PD-associated mortality for one of the sites where this substitution was seen (site 3, Table 1: 12.2%). Although higher than the average mortality observed in recently studied Norwegian Atlantic salmon sites affected by PD [9], the two sequences obtained from the study site with the highest mortality (site 2, Table 1: 26.9%) did not show this substitution. It can not be determined from this study whether any particular amino acid substitutions has had effect on the disease progression or the mortality of the affected sites, however this should be investigated further in future SAV subtype 3 sequence analyses.

The phylogenetic analyses revealed the presence of two clusters in the phylogenetic tree (Figure 1). Due to the low divergence between the sequences in the upper and lower clusters of the phylogenetic tree, the use of the term branch has been avoided. When comparing the sequences from the upper and lower clusters, a maximum of six nucleotide substitutions and four amino acid substitutions were detected. The upper cluster consists of 11 study sequences and two GenBank obtained sequences (previously found to form a separate cluster to other analysed sequences [15]), which all show the serine to proline substitution at E2 position 206. This group consists of sequences from Finnmark (sites 11 and 12, Table 1) and Troms (site 10, Table 1) together with six sequences from one site in Hordaland (site 3, Table 1) obtained in 2007 and 2009. The other three sequences from 2007 and 2009 obtained from this site (site 3, Table 1) grouped together with the remaining sequences in the lower cluster. This lower cluster also contained sequences originating from both the endemic and the non-endemic regions. One sequence from site 2 (SAVH07-2(2), Table 1) within the lower cluster separates to a certain degree from the remaining sequences. This sequence represents the site showing the highest recorded site mortality level in a recent cohort study, although no conclusion on the significance of this can be made. Sequences obtained from each site generally clustered close together. The exception to this was sequences from site 3 (Table 1) where sequences from both outbreaks (2007 and 2009) clustered in both the upper and the lower clusters. Any epidemiological interpretation of for example site-specific agent origin has proven difficult due to the high degree of similarity seen amongst the studied SAV subtype 3 sequences.

## Conclusions

It can be concluded that the analysed sequences represented only a single subtype; however some of the observed sequence divergence was higher than that previously reported by other researchers. The phylogenetic analyses confirmed that Norwegian SAV sequences can be separated into two clusters, although the differences between the two clusters were limited up to six nucleotides and four amino acids. In the future it would be desirable with larger scale, full length sequence analyses in order to enable complete sequence divergence analyses, together with investigations into the effect of particular amino acid substitutions in field outbreaks and epidemiological investigations on agent origin and spread.

## Competing interests

The authors declare that they have no competing interests.

## Authors' contributions

MDJ planned the study, performed the sequencing work and sequence analysis, and drafted the manuscript. BG: participated in the planning of the study, the sequencing work and the drafting of the manuscript. IM participated in the planning of the study and the sequencing work. JB performed the phylogenetic analyses. All authors read and approved the final manuscript.
